# Maxillomandibular myxoma in neonates

**DOI:** 10.1590/S1808-86942010000500026

**Published:** 2015-10-22

**Authors:** Adriano Santana Fonseca, Vanessa Rolim Barreto Cavalcante, Anderson Castelo Branco

**Affiliations:** 1 ENT-HNS - HC/UNICAMP. Maxillo-Facial Surgeon - Aborl/SBCP/SBCCP. Assistant Professor of Head and Neck Surgery - Santa Casa de Salvador - Hospital Santa Izabel. Full Professor of Head and Neck Anatomy - UNIME. Dysphagologist and Head and Neck Surgeon NOEV/Hospital da Bahia; 2 3rd-year Resident Physician in ENT - Hospital Santa Izabel da Santa Casa de Misericórdia da Bahia; 3 Preceptor of Maxillo-Facial Surgery at the ENT-HNS Residency Program - Santa Casa de Misericórdia da Bahia. Coordinator - Department of Maxillo-Facial Surgery - ABORL-CCF

**Keywords:** jaw abnormalities, myxoma, jaw neoplasms

## INTRODUCTION

Myxomas are rare benign mesenchymal tumors, made up of undifferentiated star-shaped cells in mucoid matrix^1^,^2^,^3^. Usually, its fibrous capsule is incomplete and infiltrates the adjacent tissue. It may involve the heart, bones, skin, aponeurotic and muscle-skeletal tissues^4^. They rarely involve the head and neck, but rather the mandible and maxilla 1. They have slow and symptomatic growth patterns; however, they are locally invasive and can grow to significant proportions. The enlargement of the affected region is frequently the reason for the patient to look for medical help. Tooth mobility can be seen and arises from alveolar changes^1^.

This neoplasia is more frequent among young patients, those between 10 and 29 years, being rarely found in individuals below 10 years of age^2^,^5^. There is a mildly higher prevalence in women, in a 1.75:1 ratio^2^. Contrary to what happens to most benign tumors, myxomas are not encapsulated and their apparent clinical and radiological limits may not represent their true limits seen upon histology^6^.

Differential diagnosis must be made between: odontogenic fibroma, ameloblastoma, dentigerous cyst, fibrous dysplasia, giant cell central granuloma, osteosarcoma and chondrosarcoma^6^.

Myxomas have a mean recurrence rate of about 25% and, the greater the surgical aggressiveness, the lower the recurrence rate^2^. The post-operative follow up of these patients is indefinite, but recurrences happen more commonly in the first two years^6^.

## CASE PRESENTATION

A male, 1-year old patient, born in Salvador/BA, with a history of a progressive mass increase in the right mandible and left maxilla, since 15 days of life, already evolving with facial deformity.

Upon physical examination, the oral mucosa was intact; however, with extensive enlargement of the mandible on the right side, extending all the way to the subcondylar portion to the height of the right lateral incisive.

CT scan showed a multilocular-type of radiolucent honey comb-like area in the body of the right mandible, extending all the way to the paraphysial region in the mandible ramus, causing expansion and destruction of the vestibular and lingual bone cortical areas. Such area involved the condylar and coronoid processes of the mandible and shifted the ipsilateral tooth germs. There was still a hypodense area with mineralized formations in the left maxilla alveolar process.

We carried out a previous biopsy, which diagnosis was fibroma. Since the lesion continued to expand, already involving the eruption of the first ipsi and contralateral teeth, we then chose surgical treatment. This was made up of a surgical excision of the entire mandible and the maxillary tumor, with a margin (right hemimandibulectomy + partial resection of the alveolar process of the left maxilla).

The histopathology exam of the specimen showed a tumor mass made up of a slightly acidophil myxoid mass and rare spindle-shaped cells, or cells mildly star-shaped, picnotic, with areas of typical osteogenesis, extra-tumoral and normal hematopoietic marrow. Matching signs and symptoms of myxoma of uncertain biological behavior and neoplasia-free surgical margins.

**Figure 1:**
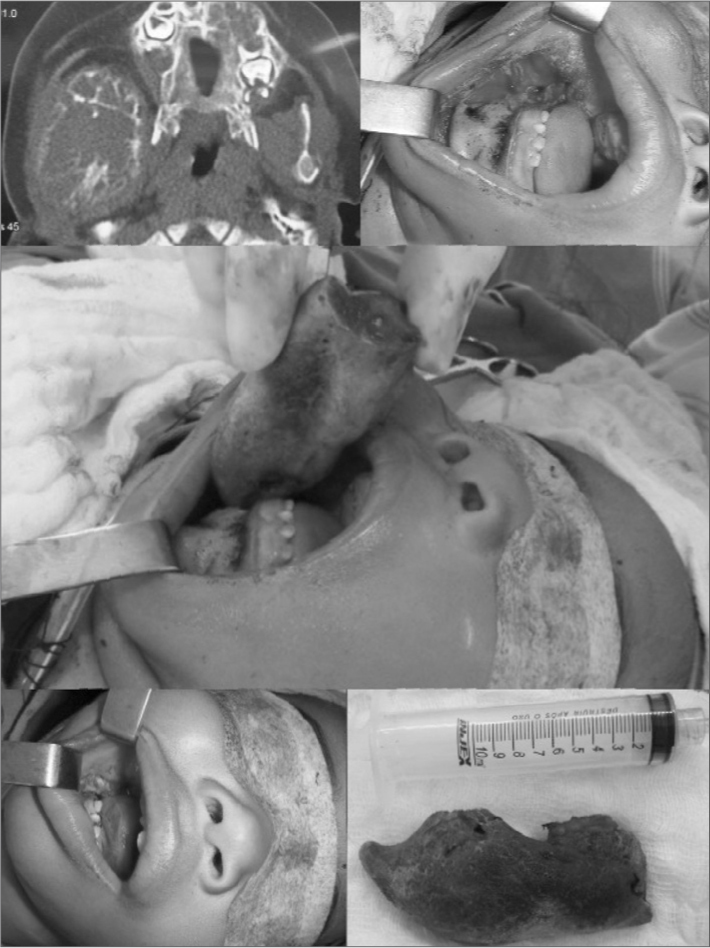
Maxillo-Mandibular Myxoma. Set of photography showing the tomographic and macroscopic aspect of the lesion and exeresis of the right-side mandible.

The hemifacial reconstruction is planned for a second procedure, with associated osteogenic distraction. (Figure 1)

## DISCUSSION AND FINAL COMMENTS

Conservative treatment of myxoma (enucleation, curettage, radiotherapy) is indicated only in proximal lesions and vital structures; however, with high recurrence rates1. Resection with proper margins is the treatment of choice to avoid recurrences1. Craniofacial reconstructions with osteogenic distraction enable a more aggressive approach and, consequently, more curative for these lesions, with the advantage of enabling an adequate oncologic control, since they reduce the need for locoregional grafts.
